# Flexible Sensing for Precise Lithium-Ion Battery Swelling Monitoring: Mechanisms, Integration Strategies, and Outlook

**DOI:** 10.3390/s25247677

**Published:** 2025-12-18

**Authors:** Yusheng Lei, Jinwei Zhao, Yihang Wang, Chenyang Xue, Libo Gao

**Affiliations:** 1 Pen-Tung Sah Institute of Micro-Nano Science and Technology, Xiamen University, Xiamen 361102, China; leiyusheng@stu.xmu.edu.cn (Y.L.); jinweizhao@stu.xmu.edu.cn (J.Z.); wangyihang2022@163.com (Y.W.); 2 Shenzhen Research Institute of Xiamen University, Xiamen University, Shenzhen 518000, China; 3 School of Aerospace Engineering, Xiamen University, Xiamen 361102, China

**Keywords:** battery swelling, flexible sensors, AI-driven analysis

## Abstract

The expansion force generated by lithium-ion batteries during charge–discharge cycles is a key indicator of their structural safety and health. Recently, flexible pressure-sensing technologies have emerged as promising solutions for in situ swelling monitoring, owing to their high flexibility, sensitivity and integration capability. This review provides a systematic summary of progress in this field. Firstly, we discuss the mechanisms of battery swelling and the principles of conventional measurement methods. It then compares their accuracy, dynamic response and environmental adaptability. Subsequently, the main flexible pressure-sensing mechanisms are categorized, including piezoresistive, capacitive, piezoelectric and triboelectric types, and their material designs, structural configurations and sensing behaviors are discussed. Building on this, we examine integration strategies for flexible pressure sensors in battery systems. It covers surface-mounted and embedded approaches at the cell level, as well as array-based and distributed schemes at the module level. A comparative analysis highlights the differences in installation constraints and monitoring capabilities between these approaches. Additionally, this section also summarizes the characteristics of swelling signals and recent advances in data processing techniques, including AI-assisted feature extraction, fault detection and health state correlation. Despite their promise, challenges such as long-term material stability and signal interference remain. Future research is expected to focus on high-performance sensing materials, multimodal sensing fusion and intelligent data processing, with the aim of further advancing the integration of flexible sensing technologies into battery management systems and enhancing early warning and safety protection capabilities.

## 1. Introduction

Lithium-ion batteries have become a key enabling technology for transportation electrification and the large-scale deployment of renewable energy systems with the whole world moving towards cleaner energy, owing to their high energy density and excellent cycling stability [1,2,3,4,5]. In recent years, the pursuit of higher energy density has driven the development of advanced battery chemistries such as high-nickel cathodes and silicon-based anodes, making volume expansion induced by electrode lattice changes and parasitic reactions increasingly prominent [6,7,8,9,10]. Such swelling behavior not only compromises the mechanical integrity of the battery but is also closely linked to performance degradation phenomena, including capacity fade and internal resistance growth; in severe cases, it may even trigger safety hazards such as thermal runaway [11,12,13,14,15].

Therefore, accurate monitoring of swelling force is essential for understanding battery failure mechanisms, improving reliability assessments and enhancing safety management [16,17,18,19]. However, existing monitoring techniques have significant limitations. Conventional rigid pressure sensors have limited flexibility and poor interfacial conformity, making them unsuitable for capturing the dynamic deformation of working batteries with high precision [20,21,22]. Other approaches, such as volumetric expansion measurement, buoyancy-based methods, strain gauges, fiber-optic sensing, radiographic imaging, optical metrology and electrochemical impedance spectroscopy, typically rely on bulky equipment or complex experimental setups. This results in low system integration and limited capability for high-dynamic, in situ monitoring under practical operating conditions [23,24,25,26,27,28,29,30,31,32].

In contrast, flexible pressure sensing technology offers outstanding mechanical compliance and stretchability, as well as high sensitivity and a broad detection range (0–10 MPa). Unlike rigid or equipment-dependent techniques, flexible sensors can intimately conform to battery surfaces and maintain continuous contact during large deformation. This enables the capture of real-time swelling signals that traditional methods fail to measure effectively. The softness and thin-film nature of flexible sensors also minimize their impact on battery packaging and allow for seamless integration at the cell and module levels. This is crucial for in situ monitoring in practical battery systems. It also provides excellent interfacial conformity and strong integration capability, offering a promising pathway for real-time, in situ monitoring of battery swelling force [33,34,35,36,37]. Advances in micro/nanofabrication techniques and the development of new functional materials have led to significant progress in the ability of flexible sensors to capture the spatial distribution of swelling, monitor dynamic expansion behavior and ensure long-term reliability in battery systems [38,39,40,41]. These advantages collectively make flexible sensing technologies a promising solution for the limitations of conventional methods, highlighting their importance in accurate, practical, and scalable lithium-ion battery swelling monitoring.

**Figure 1:**
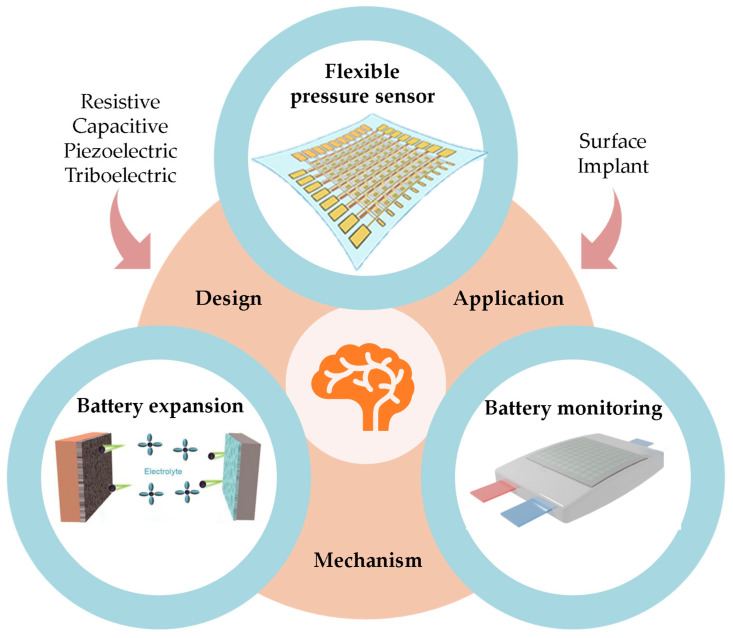
Applications and recent advances of flexible pressure sensing technology for monitoring lithium-ion battery swelling.

This work provides a systematic overview of recent advances in flexible pressure sensing technologies for monitoring swelling forces in lithium-ion batteries (Figure 1). First, we analyze the electrochemical–mechanical coupling mechanisms inside the battery and the swelling behavior that occurs during charge–discharge cycles, as well as its impact on battery performance and safety. This clarifies the key technical requirements for swelling-force monitoring. Building on this, we compare the working principles, advantages and limitations of conventional monitoring methods, including fiber-optic sensing and optical measurement techniques. Subsequently, we concentrate on the sensing mechanisms, structural design strategies, and integration approaches of flexible pressure sensors within battery systems. Furthermore, we summarize the major challenges and technological limitations from multiple perspectives, including material design, device structure, sensing performance, signal acquisition and data processing. Finally, we discuss the future development trends of flexible sensing technologies for monitoring battery swelling, with the aim of providing valuable theoretical references for research in this field.

## 2. Lithium-Ion Battery Swelling Mechanism and Monitoring Methods

The volumetric swelling behavior of lithium-ion batteries is closely associated with their complex internal electrochemical processes and interfacial reactions. This phenomenon is characteristic of charge–discharge cycling and long-term operation. In addition, this swelling can be categorized as either reversible or irreversible [22,42,43]. Reversible swelling involves periodic and recoverable volume changes during cycling, whereas irreversible swelling arises from permanent interfacial reactions or the structural degradation of electrode materials, resulting in non-recoverable volume expansion that cannot be restored even after full discharge. Therefore, accurately analyzing the mechanisms behind swelling force generation and implementing effective monitoring methods are essential for evaluating battery health, ensuring safety, and predicting service life [44,45]. This section outlines the mechanisms and significance of swelling force monitoring in lithium-ion batteries, and introduces several conventional methods for monitoring battery swelling.

### 2.1. Structure and Principle of Lithium-Ion Batteries

**Figure 2:**
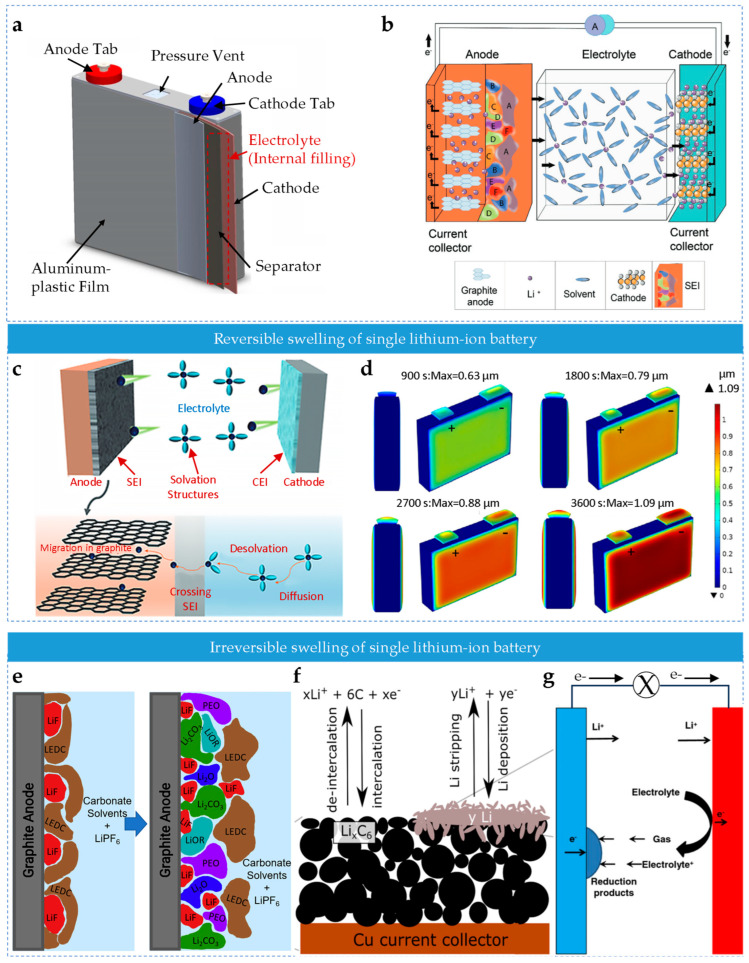
(**a**) Schematic of the typical structure of a lithium-ion battery [47]; © Elsevier, 2024. (**b**) Working principle of a lithium-ion battery [59]; © Wiley, 2023. (**c**) Lithium-ion intercalation into the graphite anode during charging [60]; © Wiley, 2021. (**d**) Reversible swelling induced by thermal expansion [61]; © Elsevier, 2020. (**e**) Irreversible swelling caused by the thickening of the solid electrolyte interphase (SEI) layer [62]; © Elsevier, 2019. (**f**) Formation mechanism of lithium deposition [63]; © ECS, 1999. (**g**) Electrolyte degradation reactions [64]; © ECS, 2016.

Lithium-ion batteries are a key device in modern energy storage. The structure and operating principle of these batteries provide the foundation for understanding the mechanisms of swelling force generation. A lithium-ion battery consists of four core components: the cathode, anode, electrolyte and separator [46,47,48] (Figure 2a). The cathode usually employs lithium-containing metal oxides, such as lithium cobalt oxide [49,50,51] (LiCoO_2_), lithium manganese oxide [52,53] (LiMn_2_O_4_) and lithium iron phosphate [54,55] (LiFePO_4_). These materials offer high specific capacity and excellent electrochemical performance, providing a stable source of lithium ions. The anode mainly comprises graphite or graphite-like carbon materials [56,57,58], which can accommodate lithium ions extracted from the cathode. The electrolyte is usually a carbonate-based solvent containing dissolved lithium hexafluorophosphate (LiPF_6_), enabling lithium-ion conduction between the electrodes. The separator is a polymer membrane with nanoscale micropores [46], which physically separates the electrodes to prevent short circuits while allowing lithium ions to pass freely and ensuring smooth ionic transport within the battery.

During charging and discharging, lithium-ion batteries undergo complex and orderly electrochemical reactions that essentially convert electrical energy into chemical energy and vice versa. Taking a lithium-ion battery with a LiCoO_2_ cathode and a graphite anode as an example [59] (Figure 2b), during charging, an external voltage causes lithium ions (Li^+^) to be released from the cathode lattice, forming Li_1−x_CoO_2_ and releasing electrons (e^−^). These migrate through the electrolyte and separator to the anode surface, where they gain electrons from the external circuit and intercalate into graphite layers to form Li_x_C_6_, thereby storing chemical energy. During discharge, the process reverses: the lithium ions deintercalated from Li_x_C_6_, migrate through the electrolyte and separator to the cathode and the electrons flow through the external circuit from the anode to the cathode. This generates current for external devices and converts the chemical energy back into electrical energy. The overall reaction can be expressed as follows (Equation (1)):

(1) $${LiCoO}_{2}+C_{6}\begin{array}{c} Charging\\ ⇌\\ Discharging \end{array}{Li}_{1-x}CoO_{2}+{Li}_{x}C_{6}$$

The microscopic charge–discharge process determines not only the energy performance of lithium-ion batteries, but also significantly influences the generation of swelling force. Based on this operating principle, we systematically analyze battery swelling mechanisms from the perspectives of reversible and irreversible expansion. This provides theoretical guidance for developing and applying swelling monitoring technologies.

### 2.2. Mechanism of Swelling Force Generation

#### 2.2.1. Expansion Induced by Anode Lithiation

During the charge–discharge cycle of a lithium-ion battery, the intercalation and deintercalation of lithium ions between the cathode and anode materials are key factors contributing to reversible swelling [65,66], primarily at the anode. In the case of the commonly used graphite anode [67], lithium ions deintercalated from the cathode during charging, migrate through the electrolyte and separator, and then intercalate into the graphite layers. This process increases the interlayer spacing of graphite and alters its lattice structure, which leads to both microscale and macroscale expansion [68,69] (Figure 2c). Graphite has a typical layered structure, with individual layers bonded by weak van der Waals forces. This enables lithium ions insertion into graphite to form Li_x_C_6_.

During discharge, the lithium ion deintercalated and returns to the cathode. This allows the graphite lattice to undergo contraction and the anode volume to shrink [60]. This reversible expansion–contraction behavior constitutes the fundamental mechanical origin of swelling force generation under normal operating conditions and serves as the starting point for understanding more complex swelling mechanisms.

#### 2.2.2. Thermal Expansion

In addition to electrochemical intercalation, temperature variation during battery operation introduces an important reversible swelling mechanism through thermal expansion. During charge–discharge cycling, heat generated increases cell temperature and induces thermal expansion in battery components [70]. A critical issue is the significant mismatch in thermal expansion coefficients between materials [71,72,73]. For instance, the thermal expansion coefficient of a graphite anode is around (3.9–4.2) × 10^−6^/°C, whereas that of an aluminum current collector can be as high as 23.6 × 10^−6^/°C. Such disparities generate considerable thermal stress within the cell, particularly under high-rate or high-temperature operation [43,74].

Although thermally induced expansion is reversible [75,76], sustained thermal stress can damage the structural integrity of electrodes, degrade contact between active materials and the current collector, increase interfacial resistance and ultimately reduce battery efficiency and energy density. Furthermore, non-uniform thermal expansion can lead to electrode bending or separator wrinkling, thereby altering lithium-ion transport pathways and potentially inducing localized current density anomalies [77,78]. These effects provide a critical link between reversible thermal swelling and subsequent irreversible degradation processes.

Multiphysics simulations clearly visualize thermal expansion behavior under various operating conditions [61] (Figure 2d). These insights provide important guidance for optimizing thermal management strategies and structural design, thereby improving overall battery performance and safety.

#### 2.2.3. Solid Electrolyte Interphase Thickening

While reversible swelling dominates under ideal conditions, repeated cycling gradually activates irreversible swelling mechanisms, among which SEI layer evolution is one of the most critical. During the initial charging of a lithium-ion battery, a series of complex electrochemical reactions occur between the anode and the electrolyte. This results in the formation of a solid electrolyte interphase (SEI) layer on the surface of the anode [79,80]. For carbonate-based electrolytes containing lithium hexafluorophosphate (LiPF_6_), the decrease in anode potential triggers the reduction of components. For example, ethylene carbonate (EC) undergoes a two-electron reduction to form lithium carbonate (Li_2_CO_3_) and organic lithium compounds [81,82,83], as shown by the following reaction (Equation (2)):

(2) $$2EC+2{Li}^{+}+2e^{-}\to {Li}_{2}{CO}_{3}+{\left({CH}_{2}{OCO}_{2}Li\right)}_{2}$$

These reduction products gradually deposit on the anode surface to form the SEI layer. Ideally, the SEI layer is ionically conductive yet electronically insulating, allowing lithium ions to pass through for subsequent electrochemical reactions while preventing electron transport [84,85]. This inhibits further electrolyte decomposition and protects the anode material. However, during cycling, the volume of the anode active particles changes due to lithium-ion intercalation and deintercalation, which induces mechanical stress on the SEI layer [62,86,87]. With repeated cycling, the SEI layer gradually cracks, exposing fresh anode material to the electrolyte and triggering additional reduction reactions. This leads to continuous thickening of the SEI layer (Figure 2e).

This progressive SEI growth marks a transition from reversible swelling to irreversible swelling, consuming active lithium and increasing internal resistance [88,89,90], thereby degrading battery efficiency and lifetime.

#### 2.2.4. Lithium Plating

Under more severe or non-ideal operating conditions, lithium plating emerges as another dominant source of irreversible swelling and safety risk. Lithium plating may occur on the anode surface of lithium-ion batteries under specific operating conditions, such as overcharging, high-rate charging or low temperatures. This represents a key factor in irreversible battery swelling [91,92]. During overcharging, lithium ions density exceeds the anode’s intercalation capacity, some of the ions are reduced directly to metallic lithium on the anode surface.

Microscopically, the formation of lithium deposits is closely related to the reduction kinetics of lithium ions on the anode surface and the structure of the electrode material [42,93,94]. Under abnormal conditions, lithium ions that cannot successfully intercalate accumulate at active sites on the anode surface and are reduced to lithium atoms. Gradually, these form metallic lithium particles. With continued deposition, these particles grow into dendritic lithium structures [63] (Figure 2f), whose sharp tips can penetrate the separator and cause internal short circuits, thereby posing a severe safety risk.

Due to the difference in density between metallic lithium and the anode material, the deposition of lithium occupies additional space, resulting in an increase in macroscopic thickness. Simultaneously, parasitic reactions between lithium metal and electrolyte further accelerate irreversible degradation [63,95].

#### 2.2.5. Electrolyte Decomposition

Besides interfacial and deposition-driven mechanisms, electrolyte decomposition is another important pathway that leads to irreversible swelling through gas generation. This degradation is primarily caused by impurities and moisture in the electrolyte, as well as by internal electrochemical reactions [96,97]. For instance, the presence of water (H_2_O) in the electrolyte causes lithium hexafluorophosphate (LiPF_6_) to undergo hydrolysis, according to the following reaction (Equation (3)):

(3) $$LiPF_{6}+H_{2}O\to LiF+POF_{3}+2HF$$

The generated hydrofluoric acid (HF) is highly corrosive, reacting with electrode materials and the SEI layer, damaging the internal structure of the battery and potentially triggering additional side reactions [98,99]. Furthermore, during electrochemical reactions, the strong oxidative nature of the cathode at high voltages and the strong reductive nature of the anode at low potentials can decompose electrolyte solvents and lithium salts, producing gases such as carbon dioxide (CO_2_) and hydrogen (H_2_) [64,100,101,102]. The accumulation of these gases raises internal pressure (Figure 2g), eventually leading to irreversible swelling.

Table 1 summarizes the types and characteristics of different battery swelling mechanisms, highlighting that accumulation of swelling force is a key factor affecting battery safety, performance, and lifespan. Therefore, efficient monitoring of swelling force is of critical practical importance. From a safety perspective, thermal runaway can be triggered by irreversible swelling due to SEI layer rupture and volume expansion caused by lithium plating [12,103,104]. This means that real-time monitoring of swelling force is essential for early warning. From a performance optimization perspective, tracking reversible swelling induced by lithium-ion intercalation/deintercalation provides guidance for electrode design and electrolyte formulation. This enhances both the rate capability and capacity stability. From a lifetime assessment perspective, the extent of swelling force accumulation correlates closely with cycle life [8,9,15,105]; long-term monitoring can reveal the relationship between swelling characteristics and battery ageing, enabling the accurate prediction of remaining battery life. Therefore, monitoring strategies capable of capturing both reversible and irreversible swelling under complex operating conditions are essential for bridging fundamental mechanism studies and practical battery applications.

### 2.3. Methods for Swelling Monitoring

Existing battery swelling monitoring methods can be broadly classified as contact-based or non-contact, depending on the measurement approach. Contact-based methods obtain local or global deformation information through mechanical contact, bonding, or embedded sensing elements. In contrast, non-contact methods use optical or radioactive imaging to track changes in external geometry or internal structure remotely. Notably, in most existing studies, traditional techniques directly measure swelling displacement or strain, while swelling force is typically an equivalent mechanical quantity determined from displacement/strain signals under specific structural constraints and mechanical model assumptions. Although these two quantities are physically distinct, they are closely linked through mechanical relationships in practical measurements. The following sections will systematically introduce the measurement principles, applicable scenarios, and respective advantages and limitations of these methods.

#### 2.3.1. Dilatometry Method

**Figure 3:**
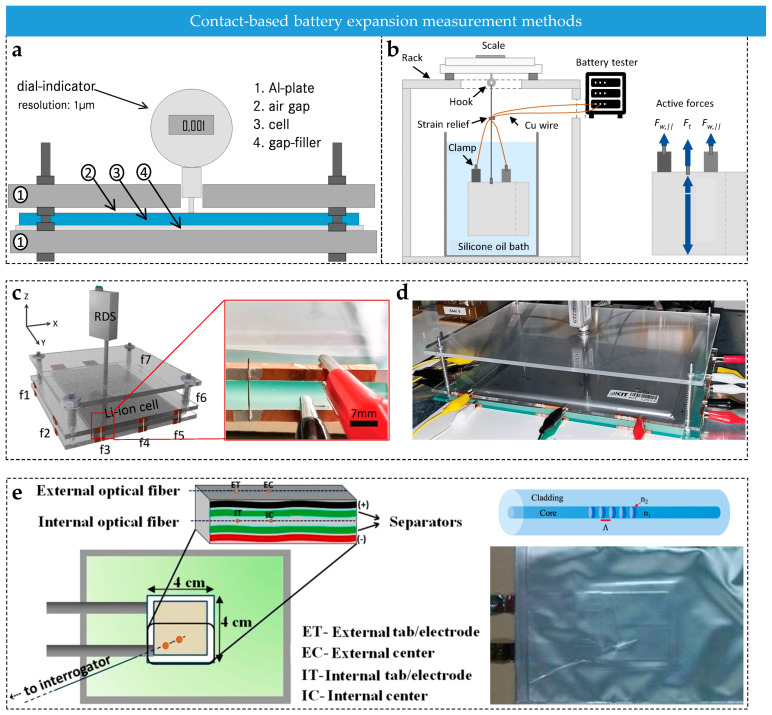
(**a**) The dilation method [109]; © Elsevier, 2014. (**b**) The buoyancy method [114]; © ECS, 2023. (**c**) The strain-based method, with an inset showing the arrangement of strain sensors. (**d**) The image of the strain-based testing setup [115]; © Wiley, 2023. (**e**) Fiber Bragg grating (FBG) sensors method. The inset on the right shows the FBG sensor and a battery integrated with embedded FBG sensors [116]; © MDPI, 2016.

The dilatometry method is a contact-based technique that measures battery swelling by tracking thickness or volume changes using high-precision displacement sensors [106,107,108]. As shown in Figure 3a, the battery is fixed to a testing platform and a micrometer is used to record displacement data on the battery surface in real time with a resolution of up to 1 μm [109]. Since swelling force is closely related to the elastic deformation of electrode materials and casing stiffness, displacement signals are converted into force values using Hooke’s law (F=k·ΔL, where k is the battery’s effective stiffness and ΔL is thickness change) or finite element simulation models [110,111].

The advantages of this method include simplicity and low cost, making it suitable for conventional conditions. However, it only provides overall swelling data and may introduce errors when inferring force from strain [112,113].

#### 2.3.2. Buoyancy Method

The buoyancy method is based on Archimedes’ principle and enables the indirect monitoring of battery volume expansion by measuring the changes in buoyant force of a submerged battery in an inert liquid [117,118,119]. As demonstrated in Figure 3b, the battery is completely submerged in a thermostatic inert medium, such as silicone oil, and a high-precision electronic balance (0.1 mg resolution) records the buoyant force changes in real time. The volume change is calculated using the formula ΔV=Δm/ρ and then converted into swelling force through a mechanical model [114]. The key of this method lies in eliminating the influence of liquid viscosity and surface tension on the measurement [117].

Although this method enables indirect measurement of total volume change, it requires well-sealed batteries and may affect heat dissipation. This makes continuous in situ monitoring challenging [114,117].

#### 2.3.3. Strain-Based Method

The strain-based method enables indirect monitoring of battery swelling force based on the piezoresistive effect [120,121]. The measurement principle is shown in Figure 3c. Strain gauges are attached to the stress-bearing parts of the battery fixture using conductive silver paste [115] (or directly to the battery surface [122,123]). When the battery expands, the minute deformation of the fixture is transmitted to the strain gauge. The strain is then combined with the mechanical relationship between expansion volume and stress to calculate swelling force. Figure 3d shows a photograph of the testing setup.

This method offers high local strain monitoring capability (resolution up to 1 με) at moderate cost. However, it also has technical limitations, including restriction of real battery deformation by adhesive curing, temperature drift requiring compensation and potential structural damage from embedded installation [124,125]. Optimization is required for practical applications depending on specific scenarios.

#### 2.3.4. Fiber Optic Sensor Method

In fiber optic sensing, fiber Bragg grating (FBG) sensors are widely used for monitoring battery swelling due to their unique wavelength modulation characteristics [126,127] (Figure 3e). When broadband light enters the FBG, light that satisfies the Bragg condition is reflected, forming a characteristic Bragg wavelength $\lambda_{B}$ (Equation (4)):

(4) $$\lambda_{B}=2n_{eff}\cdot \Lambda$$

**Figure 4:**
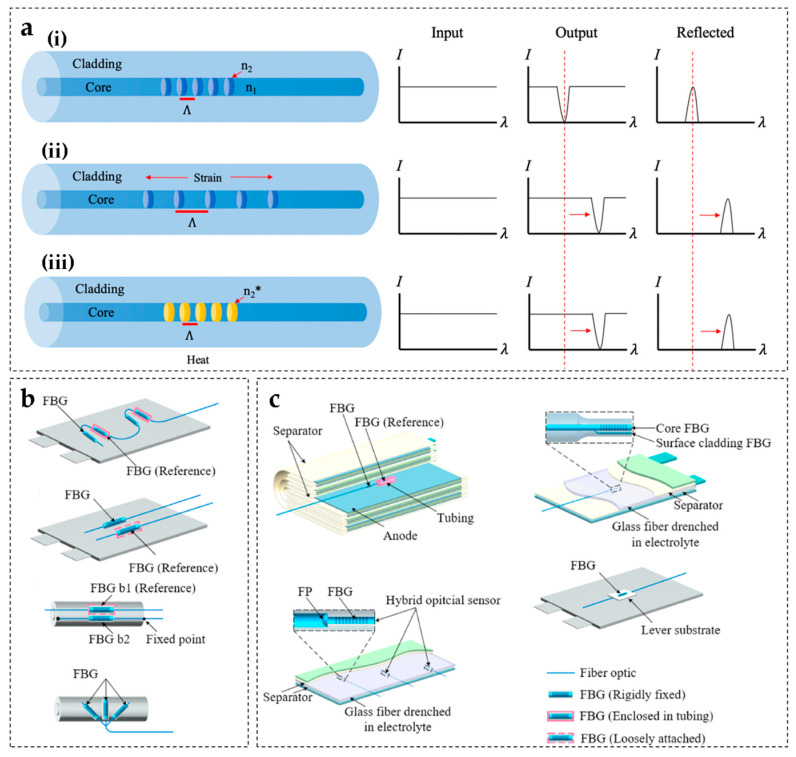
(**a**) Working principle of fiber Bragg grating (FBG) sensors. (i) A FBG with no strain and ambient heat will have a specific output and reflected profile; (ii) As strain is applied the grating spacing will change causing a shift in the output and reflected profile; (iii) And if heat is applied the effective refractive index of the gratings ($n_{2}*$) will change causing a shift in the output and reflected profile and changes in the spacing between the grating will also occur due to thermal expansion [130]; © MDPI, 2021. Battery swelling testing scheme with FBG sensors (**b**) externally attached and (**c**) embedded internally [131]; © Elsevier, 2021.

During battery swelling, the grating attached to the battery surface or embedded within the internal structure undergoes periodic changes in Λ or $n_{eff}$, resulting in a shift in $\lambda_{B}$ ($\Delta \lambda_{B}$) (Figure 4a). The wavelength shift $\Delta \lambda_{B}$ is measured using a wavelength interrogator and strain is calculated using the strain sensitivity coefficient $K_{\varepsilon }$ [128,129,130], as shown in Equation (5):

(5) $$\varepsilon =∆\lambda_{B}/\left(K_{\varepsilon }\cdot \lambda_{B}\right)$$

The swelling force is then derived through a mechanical model of the battery.

In practical applications, FBG sensors can be arranged externally (Figure 4b) or embedded internally (Figure 4c). External attachment is suitable for routine monitoring [116,132], while internal embedding places the sensor between the battery separator and the electrodes, enabling direct measurement of internal strain fields [130,131,133].

However, although FBG sensing technology shows great potential for battery monitoring, there are challenges to its application: high-precision wavelength interrogation equipment is costly, embedded sensors require strict protection against electrolyte corrosion, and the quality of the coupling between the sensor and the battery interface directly affects measurement accuracy [132,134,135]. These factors must be carefully considered in practical applications.

#### 2.3.5. Radiation Method

Radiation-based techniques such as X-rays and neutron beams enable non-destructive monitoring of internal battery swelling [136].

**Figure 5:**
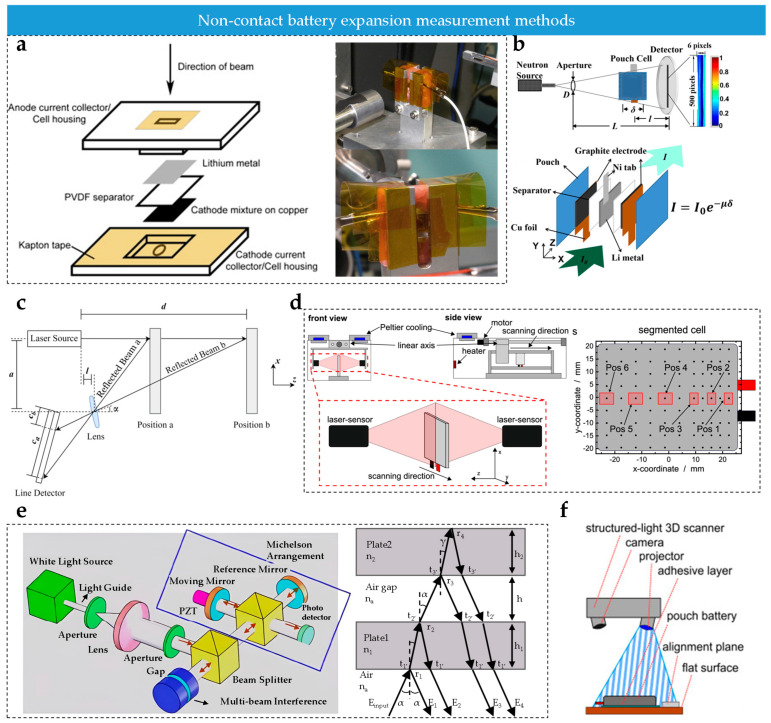
(**a**) X-ray measurement system. The inset shows the corresponding experimental setup [137]; © Elsevier, 2013. (**b**) Principle of neutron imaging [147]; © ACS, 2016. (**c**) Principle of one-dimensional laser triangulation [151]; © Elsevier, 2016. (**d**) Principle of two-dimensional laser triangulation. The inset shows the distribution of measurement points on the cell surface [150]; © MDPI, 2024. (**e**) Principle of white-light interferometry. The inset highlights the modulation of the interfering light beams [157]; © OSA, 2009. (**f**) Principle of camera-based 3D imaging [159]; © Wiley, 2020.

The X-ray method relies on the penetration and imaging characteristics of X-rays (Figure 5a) [137,138,139]. There are two main techniques:

(1) X-ray computed tomography (XCT) [140,141]. A cone-beam X-ray source penetrates the battery and the detector acquires projections that are reconstructed into a three-dimensional structure (with resolutions up to 5 μm), allowing direct observation of electrode thickness variation, particle fracture and other internal morphological changes.
(2) X-ray diffraction (XRD) [7,142]. By analyzing shifts in the diffraction peaks of the electrode crystal structure, changes in the lattice parameters and thus microscopic strain can be quantified.

Both techniques must be coupled with the elastic modulus of the electrode materials and the casing constraints in order to convert microscopic strain into macroscopic swelling pressure via the mapping relationship between strain and expansion force. A key concern is avoiding radiation-induced damage to active materials during prolonged X-ray exposure.

While X-ray-based methods provide visual insight into internal swelling mechanisms, they are limited by high equipment costs, long scanning times, an inability to capture millisecond-scale transient swelling and incompatibility with large cells [143,144].

Neutron imaging is another advanced radiation-based technique that offers unique advantages for characterizing internal batteries. Compared with X-ray imaging, neutrons are more sensitive to light elements such as lithium and can penetrate heavy metals more easily [145,146,147], enabling clear visualization of lithium distribution within electrodes (Figure 5b). Due to the strong interaction between neutrons and lithium, this method can accurately track the spatiotemporal evolution of regions with a high lithium concentration, providing valuable insight into lithium-ion transport behavior. However, neutron sources require specialized facilities, leading to high cost and limited accessibility [147].

#### 2.3.6. Optical Imaging

Optical methods are easier to deploy than radiographic techniques. Common optical approaches include laser triangulation, interferometric measurement and 3D optical imaging.

Laser triangulation reconstructs one-dimensional or two-dimensional swelling profiles by analyzing the displacement of a laser spot on the battery surface [148,149]. Due to its small spot size and high-precision sensors, this method can achieve spatial resolution at the micrometer level.

However, a fundamental trade-off exists between field of view and resolution: in one-dimensional imaging, high sampling rates are possible because the system is mainly limited by the camera exposure time. The setup is relatively simple, consisting of a laser source, lenses and a line-array detector [150] (Figure 5c). The two-dimensional configuration extends the one-dimensional method by using array scanning or multi-point acquisition to realize full-surface deformation mapping [151] (Figure 5d).

In the ideal optical model, the imaging position $C_{d}$ on the line-array detector is a non-linear function of the distance d [148,151,152,153,154] as shown in Equation (6), determined by parameters such as the focal length f, the detection angle α, the incident offset a and the reference distance l.

(6) $$d=\frac{f}{\cos\alpha }(1+\frac{\sqrt{a^{2}+(a\tan\alpha -\frac{f}{\cos\alpha })}}{C_{d}})-a\tan\alpha -l$$

Optical interferometry is based on the principle of light interference, enabling non-contact measurements with nanometer-level precision [155,156]. In white-light interferometry [157] (Figure 5e), the incident beam is split into a reference path and a measurement path. The reference path is reflected by a mirror, while the measurement path is reflected by the battery surface. The two paths are then recombined to form interference fringes. Battery swelling alters the optical path of the measurement beam, shifting the fringes. Fourier-based image processing is then used to reconstruct micro-scale surface deformation with a resolution of up to 0.1 nm. Maintaining lighting stability is crucial to avoid distortion of the fringes due to stray light [156,158].

Three-dimensional (3D) optical scanning applies triangulation with projected coded patterns to capture deformed light fields, and 3D reconstruction algorithms extract high-precision surface point-cloud data [69,159,160,161] (Figure 5f). Therefore, by comparing 3D profiles at different cycling stages, volumetric expansion and localized deformation can be precisely quantified, providing key data for swelling-force analysis. While the technique offers intuitive visualization and full-field measurement, it struggles with real-time monitoring of dynamic processes. Reflective or contaminated surfaces may introduce errors, requiring surface treatment or algorithmic correction [160].

#### 2.3.7. Electrochemical Impedance Spectroscopy

Electrochemical impedance spectroscopy (EIS) provides an effective indirect approach for monitoring battery expansion by correlating internal impedance characteristics with structural evolution [142,162]. During cycling, changes in the volume of the electrode materials alter pore structures, interfacial contact conditions and ion transport pathways. These structural variations directly impact key parameters such as charge-transfer resistance and Warburg diffusion impedance [163,164]. By establishing quantitative relationships between impedance features and expansion behavior, the battery’s swelling state can be tracked continuously.

EIS has several advantages, including non-invasiveness and operational simplicity. Furthermore, it can also simultaneously capture electrochemical states and mechanical deformation, making it suitable for long-term online monitoring. However, the method is sensitive to temperature fluctuations and contact resistance, has limited spatial resolution and relies on accurate equivalent-circuit modeling for reliable quantification. These limitations restrict its applicability under complex operating conditions [165,166].

Table 2 provides a systematic summary and comparative analysis of conventional techniques for monitoring battery swelling. Existing methods exhibit inherent limitations: among contact-based approaches, the strain-gauge method is susceptible to temperature drift, while fiber-optic sensors suffer from relatively high cost. Among non-contact approaches, X-ray techniques require expensive instrumentation; optical interferometry requires highly stable environmental conditions; and 3D scanning technologies struggle to capture real-time dynamic deformation. Consequently, it remains challenging for traditional methods to simultaneously achieve high accuracy, real-time capability, in situ applicability and cost-effectiveness.

These limitations are particularly evident in transient monitoring during high-rate cycling and long-term stability under complex operating conditions, and the demand for coupled micro–macro deformation characterization. Such challenges highlight the need for advanced monitoring solutions capable of overcoming the constraints of conventional methods. In this context, flexible pressure-sensing technologies emerge as a promising alternative. With their intrinsic mechanical flexibility, excellent interfacial conformity and high sensitivity to mechanical stimuli, flexible sensors offer distinct advantages and have the potential to advance swelling-force monitoring towards greater precision, in situ implementation, dynamic measurement and integrated system design.

## 3. Flexible Pressure Sensors

Flexible pressure sensors are a new type of device that uses flexible materials, such as polydimethylsiloxane (PDMS) and polyimide (PI), as substrates or active sensing elements [34,37,167,168]. The underlying operating principle of these devices is that external mechanical stimuli are converted into measurable electrical signals. This section will focus on the device architecture, sensing mechanisms and key performance parameters of flexible pressure sensors.

### 3.1. Composition of Flexible Pressure Sensors

**Figure 6:**
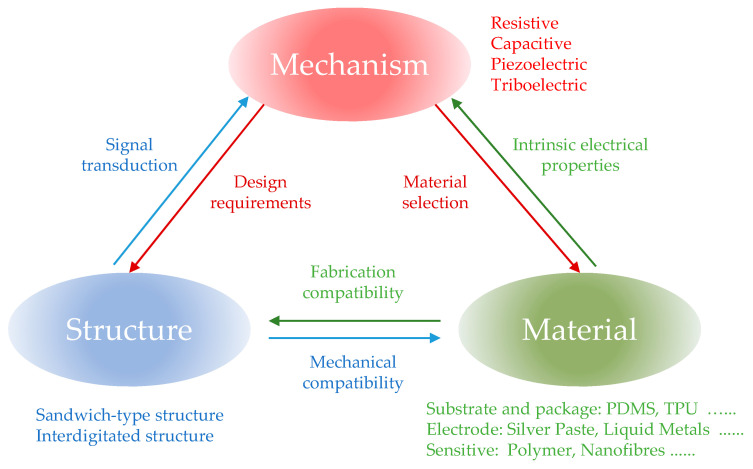
The mechanism, structure and material relationship of flexible pressure sensors.

The performance of flexible pressure sensors depends on three closely related elements: the sensing mechanism, structural design, and material system (Figure 6). They collectively define the sensor’s overall composition and functional capabilities, establishing the foundation for accurately and reliably measuring battery swelling forces.

A flexible pressure sensor typically consists of four layers: a flexible substrate, a spacer layer, an electrode layer, and a sensing layer. The substrate provides mechanical support and conformability, enabling the sensor to adapt to curved or deformable surfaces. The encapsulation layer protects the sensor from environmental factors, such as humidity and chemical corrosion. The spacer layer ensures electrical isolation between the electrodes and reduces signal crosstalk, which contributes to measurement accuracy. The electrode layer efficiently collects and transmits electrical signals, and the sensing layer directly converts mechanical stimuli into measurable electrical responses.

The material system is essential for enabling the specific functions of each layer. Common flexible substrates include PDMS [169,170] and PI [171], which offer elasticity and durability. Electrodes are usually made of silver nanowires [172,173], conductive polymers, or graphene-based flexible conductors [174], which ensure stable electrical connectivity. The material used for the sensing layer depends on the intended mechanism. For example, semiconducting polymers are used for piezoresistive sensors [175], dielectric elastomers are used for capacitive sensors [176], PVDF is used for piezoelectric sensors [177], and porous polymers are used for triboelectric sensors [178,179,180].

Coordinating these elements—the mechanism, structure, and materials—allows for the customization of a sensor’s composition to meet practical requirements, including sensitivity, durability, and environmental stability. The following subsections discuss specific mechanisms and structural designs in detail, building upon this foundational understanding of sensor composition.

### 3.2. Mechanism and Structure of Flexible Pressure Sensors

Flexible pressure sensors convert the mechanical stress induced by battery swelling into quantifiable electrical signals, enabling the precise monitoring of battery expansion behavior. The performance is primarily determined by the choice of sensing mechanism and the configuration of structural design, both of which jointly determine the sensitivity, response time, and applicability of the sensor across different operating conditions.

#### 3.2.1. Sensing Mechanism

The sensing mechanism provides the physical basis for converting mechanical stress into electrical signals. Current mainstream mechanisms include piezoresistive (resistive) [181,182,183], capacitive [184,185,186], piezoelectric [187,188], and triboelectric types [189,190], allowing precise and reliable monitoring of battery expansion behavior.

**Figure 7:**
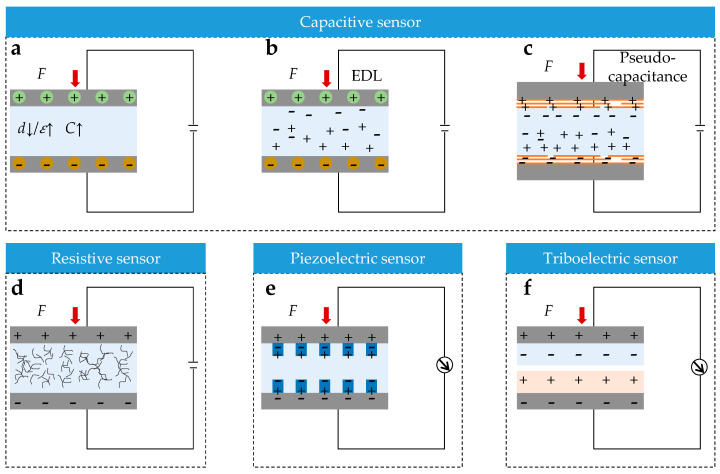
(**a**) Conventional parallel-plate capacitive, (**b**) iontronic capacitive, (**c**) pseudocapacitive, (**d**) resistive, (**e**) piezoelectric and (**f**) triboelectric sensing mechanisms.

Capacitive sensing is based on the parallel-plate capacitor model (Figure 7a), with its capacitance described by Equation (7) [191]:

(7) $$C=\frac{\varepsilon_{0}\varepsilon_{r}\cdot A}{4\pi kd}$$

where $\varepsilon_{0}$ is the vacuum permittivity, $\varepsilon_{r}$ is the relative permittivity, A is the effective electrode area, k is the electrostatic constant and d is the electrode spacing. External pressure induces capacitance variation by altering d or A.

Traditional sandwich-type structures offer good stability, but have a limited response speed. In contrast, emerging iontronic structures (Figure 7b) leverage the rapid reconstruction of the electric double layer [38,192,193,194] (EDL), enabling an ultrafast response. Furthermore, the introduction of pseudocapacitive materials, such as MXene [195,196] (Figure 7c), further enhances sensitivity, thereby enabling this mechanism to detect subtle expansion forces in small-capacity lithium batteries.

Resistive (piezoresistive) sensing relies on the piezoresistive effect (Figure 7d), where resistance is described by Equation (8) [197]:

(8) $$R=\frac{\rho \cdot L}{A}$$

where ρ is resistivity, L is length and A is cross-sectional area. The resistivity of the sensing material plays a central role in determining sensitivity and stability [198]. However, environmental humidity, temperature fluctuations and long-term mechanical fatigue can significantly degrade performance.

Piezoelectric sensing operates on the direct piezoelectric effect and converts dynamic pressure into voltage without an external power supply (Figure 7e). Its output voltage is described by Equation (9) [199]:

(9) $$V=\frac{d_{33}\cdot Ft}{\varepsilon_{0}\varepsilon_{r}\cdot A}$$

where $d_{33}$ is the piezoelectric coefficient, F is the applied force, t is the material thickness, $\varepsilon_{0}$ and $\varepsilon_{r}$ are the vacuum and relative permittivity, and A is the electrode area. However, this mechanism is not suitable for monitoring static pressure, and the piezoelectric coefficient is highly temperature-dependent [177].

Triboelectric sensing is based on the principle of the triboelectric nanogenerator (TENG) [179] (Figure 7f). Contact and separation between materials with different electronegativities induces charge transfer, and the open-circuit voltage is described by Equation (10):

(10) $$V_{oc}=\frac{\sigma \cdot d}{\varepsilon_{0}\varepsilon_{r}}$$

where σ is the surface charge density, d is the separation distance. Its major advantages are self-powered operation and a strong signal output, making it ideal for operating in harsh environments [180,200]. However, long-term stability is limited due to charge decay in the triboelectric layers and insufficient contact–separation on curved cells, which can introduce substantial measurement errors [201].

#### 3.2.2. Sensing Structure

**Figure 8:**
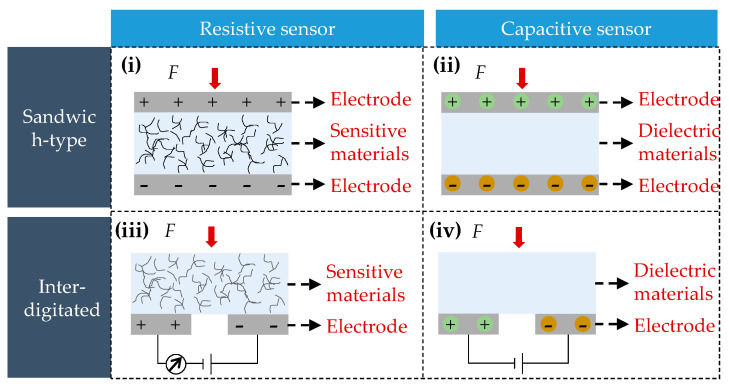
Typical structures of flexible pressure sensors. (**i**) Sandwich-type piezoresistive and (**ii**) sandwich-type capacitive sensors and (**iii**) interdigitated piezoresistive and (**iv**) interdigitated capacitive sensors.

The macroscopic architecture of flexible pressure sensors determines the way in which they integrate with batteries and capture mechanical information. As illustrated in Figure 8, there are two primary structural types: the sandwich design [41,197,202] and the interdigitated design [203,204,205].

The sandwich configuration has a vertically stacked “electrode–sensing layer–electrode” layout, which is the typical structure of capacitive and piezoresistive sensors [206]. Its good conformability, simple fabrication process and controllable thickness enable the sensor to be tightly attached to the battery surface, allowing large-area monitoring of pressure distribution. However, the presence of multiple interfaces may impact long-term interfacial stability [207].

By contrast, the interdigitated architecture creates an in-plane array of interleaving electrodes, offering high spatial resolution and excellent localized stress detection capabilities. Piezoresistive interdigitated sensors employ nanocomposite fillers in the gaps between the electrodes to precisely identify stress concentration near critical regions [208]. Thereby, capacitive interdigitated sensors rely on pressure-induced variations in the dielectric layer to regulate the effective permittivity. This allows the electrodes to function only as capacitive probes, preventing direct contact between the sensing materials and the battery. This greatly enhances long-term stability and makes the structure particularly suitable for monitoring shell expansion in cylindrical or prismatic cells under corrosive environments [209,210].

Overall, sensing mechanisms and structural design jointly define the technical foundation of flexible pressure sensors. From a functional perspective, the mechanism determines the intrinsic performance limits—for instance, piezoelectric sensors are suited to dynamic measurements, whereas capacitive sensors are better for steady-state detection. Meanwhile, the structural design ensures compatibility with various battery geometries and monitoring requirements.

### 3.3. Performance of Flexible Pressure Sensors

In the field of lithium-ion battery swelling monitoring, the selection of flexible pressure sensors must be closely matched to the battery’s operational characteristics. The sensor’s key performance metrics directly impact the reliability of monitoring in various application scenarios. Sensitivity is crucial for detecting subtle swelling signals. Specifically, during normal charge–discharge cycles, the lithiation/delithiation of electrode materials induces subtle volumetric changes, particularly during the formation process or when evaluating new materials. Therefore, high-sensitivity sensors are required to capture these micro-scale pressure fluctuations [211,212]. In addition, under extreme conditions such as overcharge, batteries may undergo severe bulging, thus necessitating the sensor with wide sensing range that spans from low to high pressures [213].

Hysteresis and stability are equally important because they influence the accuracy and reliability of swelling measurements. As the swelling force evolves dynamically during cycling, excessive hysteresis can cause a phase lag between the measured and actual pressure values, which impacts the quantification of swelling rate and peak stress [214,215]. Long-term stability is also crucial; otherwise, it becomes difficult to track the evolution of swelling behavior throughout the battery’s lifespan [216].

Response speed is critical in fast-charging scenarios. With the rapid development of high-rate charging technologies, batteries may experience sudden pressure spikes, so sensors are necessary that can capture these transient events with sufficiently fast dynamic responses [217,218]. Moreover, since the battery environment typically involves temperature fluctuations and electrolyte evaporation, robust environmental resistance is also necessary to ensure long-term measurement stability [184,219,220].

Different types of flexible pressure sensor exhibit distinct performance characteristics (Table 3). In general, capacitive sensors provide a balanced combination of sensitivity, sensing range and low hysteresis, making them widely applicable to regular swelling monitoring [221]. By contrast, Iontronic sensors benefit from electric double-layer and pseudocapacitive mechanisms, thereby achieving exceptionally high sensitivity and enabling the detection of minute swelling signals associated with lithiation processes [40,200]. Meanwhile, resistive sensors offer a wide dynamic range and excellent long-term stability, making them suitable for monitoring swelling throughout the entire battery lifecycle, from minor deformation to severe bulging [202,222]. In addition, piezoelectric sensors excel at capturing transient stresses during charge–discharge processes due to their fast response characteristics [223,224], while triboelectric sensors offer the benefit of self-powered operation, making them highly suitable for long-term off-grid monitoring scenarios [205].

Despite the significant advantages that iontronic pressure sensors offer in micro-scale swelling research, their measurement range is limited by ion transport properties, which means they cannot accommodate extremely high-pressure scenarios [225,226,227]. Their long-term stability may be affected by electrolyte evaporation and temperature changes, thus necessitating specialized encapsulation. Furthermore, given their relatively short operational lifetime, these sensors are better suited to high-precision in situ laboratory measurements than to long-term deployment in battery packs [17,228,229].

Overall, different types of flexible pressure sensor complement each other in the monitoring of lithium-ion battery swelling: iontronic sensors enable ultra-high-sensitivity detection and micro-mechanism studies, while capacitive and resistive sensors provide stable, wide-range monitoring for practical battery-pack applications. Piezoelectric and triboelectric sensors offer advantages in terms of dynamic response and self-powering, respectively. So, selecting the most suitable sensor types based on specific operating conditions enables the accurate and reliable monitoring of battery swelling behavior.

## 4. Application of Flexible Pressure Sensors in Lithium Battery Monitoring

Building on the systematic analysis of the performance and characteristics of flexible pressure sensors, a more informed and precise sensor selection strategy can be established for lithium-ion battery swelling-force monitoring. Due to their distinct advantages in terms of sensitivity, dynamic range, response time and stability, various types of flexible sensor have gradually enabled comprehensive application, ranging from laboratory-level in situ measurements to online monitoring of large-scale battery systems.

Therefore, this section focuses on their application modes across different battery types and operating scenarios. Representative case studies are summarized to demonstrate the practical application of flexible pressure sensors in monitoring settings and their contribution to battery safety assessment and mechanistic investigation.

### 4.1. Monitoring Strategy for Single Lithium-Ion Batteries

Monitoring lithium-ion battery swelling using flexible pressure sensors can be categorized into two approaches: surface-mounted sensing and embedded sensing. These strategies rely on different spatial coupling mechanisms to capture the evolution of swelling-induced mechanical stress.

**Figure 9:**
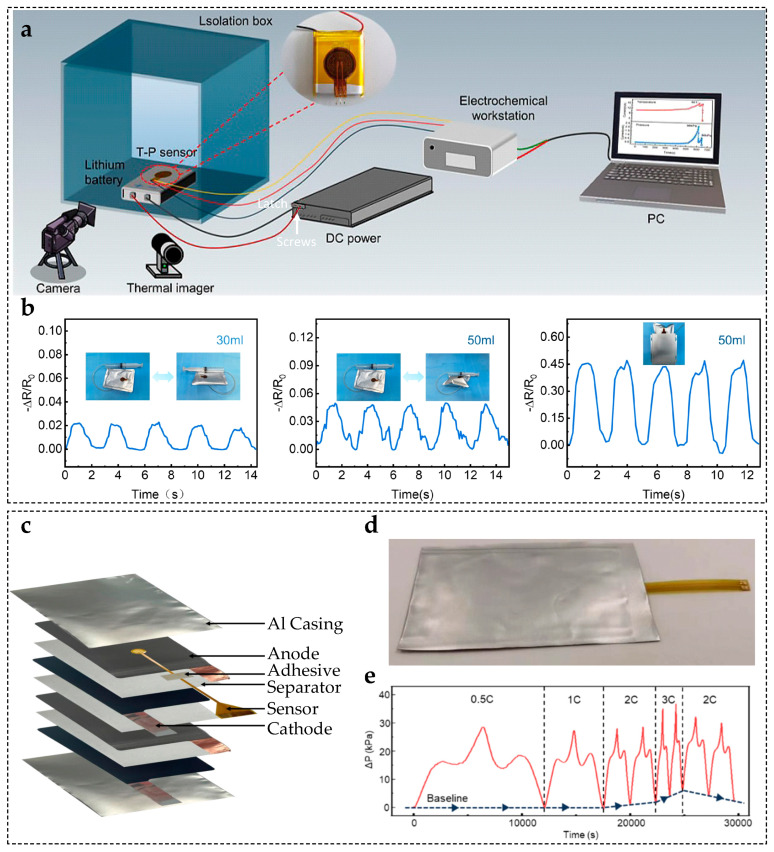
(**a**) Surface-mounted battery swelling test scheme. (**b**) Analysis of battery swelling at different expansion levels [19]; © Elsevier, 2025. (**c**) Embedded battery swelling test scheme. (**d**) Image of a lithium-ion battery with the embedded sensor. (**e**) Swelling analysis under different charge/discharge rates [237]; © The Author(s) 2025; OUP & CSPM.

In the surface-mounted approach, flexible pressure sensors are attached directly to the battery casing, enabling non-invasive monitoring of global swelling behavior [19] (Figure 9a). The reliability of this method has been validated through experimental results (Figure 9b). This method is highly suitable for practical applications due to its structural integrity, deployment flexibility and cost-effectiveness, making it ideal for integrating into existing battery systems [18,230].

Surface-mounted sensing technology has evolved from single-point detection to high-resolution, array-based monitoring. Early studies employed single-point piezoresistive sensors on pouch-cell surfaces to establish the relationship between swelling force and state of charge (SOC). Li et al. [231] proposed a real-time monitoring approach using a single-point piezoresistive sensor to record battery volume changes at various SOC. Their findings clearly demonstrated a pronounced linear relationship between swelling force and SOC, laying an important foundation for subsequent studies in this field. Building on this work, Lin et al. [232] investigated battery swelling behavior using piezoresistive sensors and showed that the sensors could accurately capture volume variations under different charging rates. This verified the structural stability of electrode materials during cycling. Barai et al. [233] employed flexible, thin-film sensors attached to the surface of a battery to study the effect of external, compressive loads on battery degradation. The study revealed pronounced capacity and power fade phenomena and clarified the evolution of compressive stress over multiple charge–discharge cycles. Furthermore, Liu et al. [234] proposed a flexible piezoelectric sensor capable of real-time monitoring and early warning of external vibrations and short-circuit events during battery operation. This approach has significant potential for enhancing safety in electric vehicle applications. More recently, Zhang et al. [19] reported a novel single-point sensor capable of dynamically monitoring the swelling process with high temporal resolution, offering a new perspective for evaluating battery performance. These early studies laid an important theoretical foundation for surface-mounted monitoring technology.

However, the limited spatial resolution of single-point sensors restricts our understanding of local deformation heterogeneity. Currently, this limitation is being addressed by a recent emphasis on sensor array development. Gan et al. [235] developed a piezoresistive array capable of distinguishing swelling differences between the tab region and the cell center. Sun et al. [16] integrated multi-sensor data with machine learning models to identify localized swelling regions during fast charging. Liu et al. [236] proposed an adaptive sensing network that dynamically adjusts monitoring strategies, demonstrating strong potential for lifetime prediction and fault diagnosis.

In contrast to surface-mounted methods, embedded sensing requires placing the sensors directly inside the battery [237] (Figure 9c). This approach imposes strict requirements regarding sensor thickness, flexibility and electrochemical compatibility. Figure 9d shows a representative embedded configuration, and Figure 9e demonstrates its effectiveness under various charge rates. The key advantage of embedded sensing is its ability to capture internal, localized swelling forces, which provide critical data for mechanistic studies [28,238].

Embedded research is committed to achieving superior electrochemical compatibility and enhanced spatial resolution. Early embedded research focused on inserting thin sensors directly into the cell stack. For instance, Guo et al. [239] embedded an ultrathin piezoelectric sensor between electrode layers, enabling real-time sensing of internal battery pressure. Subsequently, Ling et al. [240] proposed a flexible, thin-film device that can be integrated into pouch lithium-ion batteries. This device enables real-time monitoring of internal expansion-related information. This approach provides an innovative solution for battery health monitoring with low cost, high accuracy, and rapid response.

However, these early embedded approaches also revealed clear limitations. Most importantly, the sensor itself disturbs ion transport pathways, which can lead to significant capacity degradation. To minimize the invasiveness of embedded sensors on the structure and electrochemical performance of batteries, research efforts have increasingly focused on the systematic optimization of sensor miniaturization and material compatibility [18,241]. For instance, Fleming et al. [242] significantly reduced the adverse effects of sensors on electrochemical reactions and ion transport by applying Parylene C coatings and downsizing the overall device structure. Meanwhile, Gao et al. [243] designed porous or mesh-like sensor architectures that allow sufficient electrolyte infiltration to maintain ionic conductivity. This mitigates interference with the electrochemical system while preserving signal quality. These improvements have collectively alleviated sensor invasiveness and laid the foundation for continued development of embedded monitoring technologies.

A major breakthrough in recent years has been the emergence of integrated iontronic sensing [237]. This approach utilizes the electrolyte itself to form the sensing interface, eliminating the need for additional encapsulation. Experiments demonstrate that this method achieves high sensitivity to Pascal-level swelling forces, while maintaining stable operation inside the battery over extended periods. This shows its strong potential for long-term, high-precision internal state monitoring.

A comprehensive multiscale swelling-monitoring framework is formed by the combined use of surface-mounted and embedded approaches, spanning from macroscopic external deformation to microscopic internal stress analysis. As summarized in Table 4, surface-mounted sensors excel in practical, system-level monitoring and safety management, whereas embedded sensors provide the unique, in situ measurement capabilities that are essential for mechanistic investigations and advanced material evaluation.

### 4.2. Integration Strategies for Flexible Pressure Sensors in Lithium Batteries

Flexible pressure sensors can be integrated into lithium-ion batteries using different strategies, depending on the measurement target and battery structure. For surface-mounted monitoring, two common strategies are adhesive integration (fixed contact) and fixture integration (non-fixed contact). These strategies provide reliable attachment and stable signal acquisition on the battery exterior. For embedded monitoring, implant integration places sensors directly at critical internal interfaces, enabling high-quality measurements of the battery’s internal mechanical state. Each approach has its own advantages and disadvantages in terms of signal quality, mechanical compatibility, and complexity of implementation.

#### 4.2.1. Adhesive Integration and Fixture Integration Solutions

Adhesive integration (fixed contact) and fixture integration (non-fixed contact) are the primary strategies for surface-mounted monitoring of lithium-ion batteries. These approaches accommodate the diverse structural characteristics of battery modules, providing stable attachment and reliable monitoring of surface swelling. The choice of sensor integration depends on factors such as sensor placement, required contact stability, and feasibility across different battery formats.

Adhesive integration permanently bonds the sensor to the battery surface, enabling efficient mechanical load transfer and stable signal acquisition. Commonly used adhesives, such as epoxies, acrylics, and silicone-based materials, allow for precise control over modulus and thickness. This minimizes stress attenuation while maintaining electrical insulation [16,244]. This integration method offers excellent bonding reliability within the temperature range of −20 °C to 60 °C, making it well-suited for high-precision laboratory measurements [121].

By contrast, mechanical clamping integration employs an external fixture to apply a controlled preload, forming a stable yet reversible contact interface [228,245]. Through optimized clamping configurations or adaptive structures, this approach effectively accommodates changes in battery volume during cycling. Its advantages include easy installation, reusability, and non-destructive contact. These make it highly appropriate for industrial-grade online monitoring and rapid diagnostics [242,246].

In practical applications, different battery geometries are matched with different integration strategies to ensure reliable swelling characterization.

**Figure 10:**
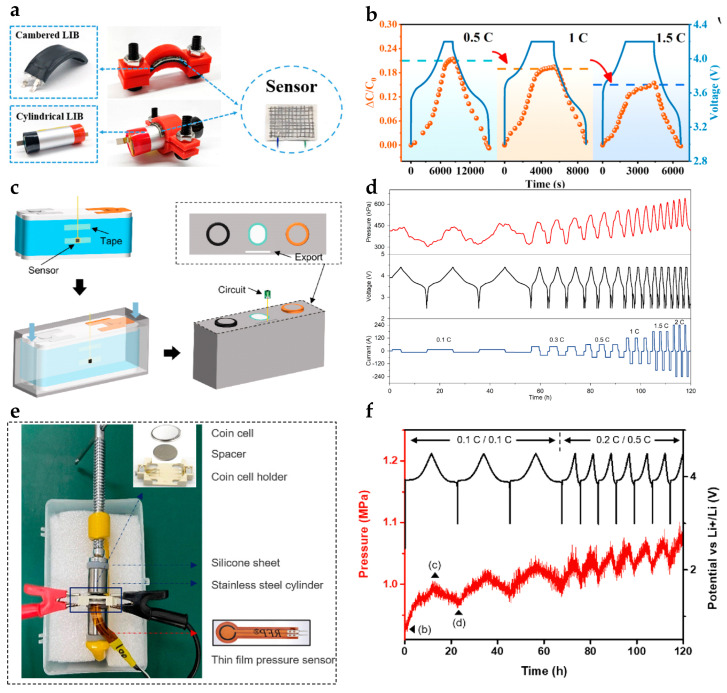
(**a**) Integration of flexible pressure sensors on the surfaces of prismatic-arched and cylindrical lithium-ion batteries. (**b**) Capacitance response of sensor under different cell charging/discharging rates [231]; © Elsevier, 2024. (**c**) Integration of flexible pressure sensors on the surfaces of prismatic lithium-ion batteries. (**d**) The relation of voltage and pressure in rates of 0.1 C, 0.3 C, 0.5 C 1 C, 1.5 C, and 2 C [247]; © Elsevier, 2023. (**e**) Surface integration scheme for coin cells and sensors. (**f**) The relation of voltage and pressure under different cell charging/discharging rates [249]; © Editorial office of Acta Physico-Chimica Sinica.

For curved batteries, such as arched and cylindrical cells, mechanical clamping is generally preferred due to its excellent geometrical adaptability. As illustrated in Figure 10a, customized fixtures can uniformly attach thin-film flexible pressure sensors to curved battery casings, preventing residual stress or uneven adhesion that could result from bonding [231]. Test results show a linear correlation between peak swelling force and charge/discharge rate (Figure 10b). This provides valuable insight into the mechanical behavior of curved batteries.

For prismatic lithium-ion batteries, which have large, flat surfaces, adhesive integration is commonly used to deploy single-point or distributed sensors to capture global swelling evolution [247,248]. As shown in Figure 10c, bonded sensors maintain stable contact throughout long-term cycling, enabling reliable monitoring of steady-state and quasi-steady-state swelling. Notably, the results demonstrate that the swelling force peaks near the end of the charging process and gradually increases with cycling (Figure 10d). This trend strongly correlates with capacity fade, thus offering an effective mechanical indicator for evaluating the health of prismatic batteries.

For the monitoring of micro coin cells, clamp-based integration is commonly adopted. In this approach, a microscale flexible piezoresistive sensor is securely attached to the surface of the coin cell using a precisely engineered testing fixture [249,250] (Figure 10e). Experimental data indicate a clear correlation between the cell’s expansion force and the applied charge/discharge rate (Figure 10f). Furthermore, the sensor can identify subtle pressure variations associated with lithium insertion and extraction. This provides valuable insight into the microscale expansion mechanisms of electrode materials.

Overall, the integration strategy for flexible pressure sensors should be selected based on the cell geometry, size constraints, and monitoring objectives. In general, adhesive bonding is well-suited for fixed configurations that require high signal fidelity and long-term stability. In contrast, clamp-based integration offers distinct advantages for multi-scenario deployment and industrial online diagnostics due to its ease of installation, non-destructive contact, and reusability [251,252,253].

#### 4.2.2. Implant Integration Solution

Implanted integrated solutions achieve direct, high signal-to-noise ratio measurements of the battery’s internal mechanical state by embedding sensors at critical interface locations during cell manufacturing. However, to ensure compatibility with the electrochemical environment while minimizing interference with ion transport pathways, the integration strategy must be customized according to battery architecture, internal space, and packaging characteristics.

**Figure 11:**
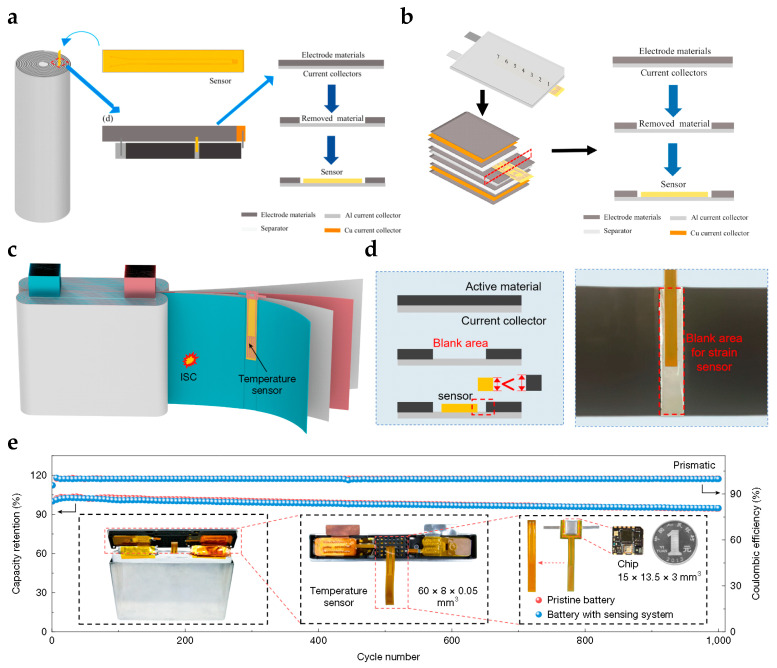
(**a**) Schematic of embedding flexible sensors into cylindrical lithium-ion batteries [251], © Elsevier, 2021, (**b**) pouch lithium-ion batteries [241], © Elsevier, 2020, and (**c**) prismatic lithium-ion batteries. (**d**) Principle of integrating a flexible sensor inside a prismatic lithium-ion battery. The inset shows the integrated image. (**e**) Analysis of aging cycling results for the prismatic lithium-ion battery embedded with a flexible sensor [18]; © Springer Nature.

For cylindrical batteries, flexible sensors in strip or sheet form can be positioned between the electrode and separator layers to dynamically monitor radial pressure within the winding core [254,255] (Figure 11a). Precise routing of sensor leads is critical to ensure extraction from the tab region does not impair cell hermeticity while maintaining signal integrity.

Pouch batteries, due to their stacked structure and aluminum-plastic packaging, offer convenient conditions for sensor placement. Sensors are typically embedded between the positive and negative electrode layers to directly record local pressure changes caused by active material expansion during charge–discharge cycles [241] (Figure 11b). This integration requires ultra-thin sensors with high electrolyte compatibility to minimize interference with ion transport and electrochemical reactions.

Large prismatic batteries have a relatively spacious interior that enables sensor placement between the winding core and casing or among different electrode groups [18] (Figure 11c). Figure 11d illustrates the principles and implementation of embedded integration, highlighting the strict requirements for sensor flexibility, packaging, and internal layout. Through miniaturization and careful material selection, optimized embedded designs ensure that the capacity degradation curve of the sensor-integrated battery closely matches that of the original battery (Figure 11e), thereby confirming minimal impact on ion transport efficiency and overall electrochemical performance.

In summary, embedded flexible pressure sensors enable high-precision in situ measurement of microscopic internal expansion forces, providing direct data support for mechanistic studies and new electrode material development, while ensuring long-term reliable monitoring without sacrificing battery performance.

Table 5 provides a systematic overview of the structural characteristics and application of the three flexible pressure sensor integration strategies. In practical applications, the selection of an appropriate integration method should be guided by the specific monitoring requirements of the cell or module. The different integration strategies vary substantially in terms of mechanical coupling quality, measurement accuracy, and environmental adaptability. These factors significantly influence the final test results. Therefore, properly matching the integration strategy is essential for reliable swelling monitoring and effective safety diagnostics.

### 4.3. Monitoring Strategy for Lithium-Ion Battery Module

Monitoring the expansion force of lithium-ion battery modules requires considering inter-cell interactions and overall structural stability. Thus, two typical strategies have emerged for flexible pressure sensors: single-point monitoring and array-based monitoring.

**Figure 12:**
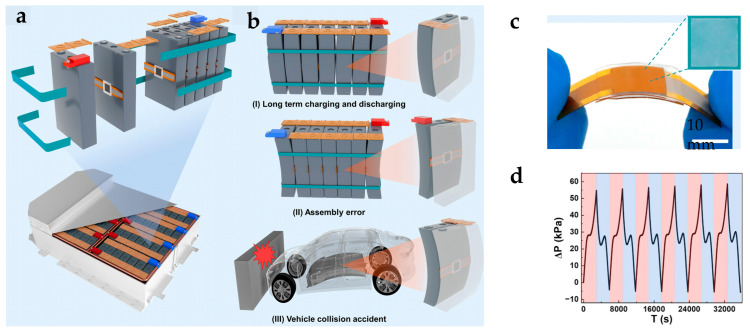
(**a**) Schematic of the integration of a flexible pressure sensor and a battery module. (**b**) Schematic diagrams of cell deformation under different operating scenarios. (**c**) Photograph of the flexible pressure sensor. (**d**) Expansion analysis of the battery module [17]; © Wiley, 2025.

In single-point monitoring, sensors are placed at critical stress points within the module, whereby local signals reflect the overall expansion trend [17] (Figure 12a). These sensors typically have ultra-thin PI substrates and composite sensitive layers to minimize their impact on the module’s structure and thermal management. Utilizing deformation schematics (Figure 12b) and sensor devices (Figure 12c), researchers acquired pressure data during charge–discharge cycles and long-term aging. Then they fitted the results to quantify variations in module expansion force with operating conditions (Figure 12d).

Recent studies have demonstrated the practical value of single-point monitoring. For instance, Chen et al. [247] deployed iontronic flexible sensors between cells in a prismatic module. They observed stepwise increases in expansion force over cycling. Low-temperature peak forces exceeded room temperature by over 40%, clearly showing the effects of temperature on module mechanics. Yin et al. [256] developed a structurally decoupled multimodal sensor that integrates piezoelectric and resistive temperature sensing. This enables simultaneous monitoring of temperature and expansion. In pouch cell tests, this sensor successfully detected overheating and expansion faults, highlighting the potential of single-point monitoring for health diagnostics. Chang et al. [257] investigated the dynamic behavior of lithium-ion battery packs under various operating conditions using flexible sensing technology combined with an embedded monitoring strategy. The results revealed that under dynamic operating conditions, the deviation between the sensor-measured internal temperature and simulation predictions could be as high as 2.6 °C. This highlights the limitations of model-based estimation and underscores the necessity of real-time, in situ battery monitoring.

However, single-point approaches have limited spatial coverage, which makes it difficult to capture regional variations in module expansion. This can lead to misjudgments in non-uniform deformation scenarios and limits the ability to localize faults [258,259,260,261].

**Figure 13:**
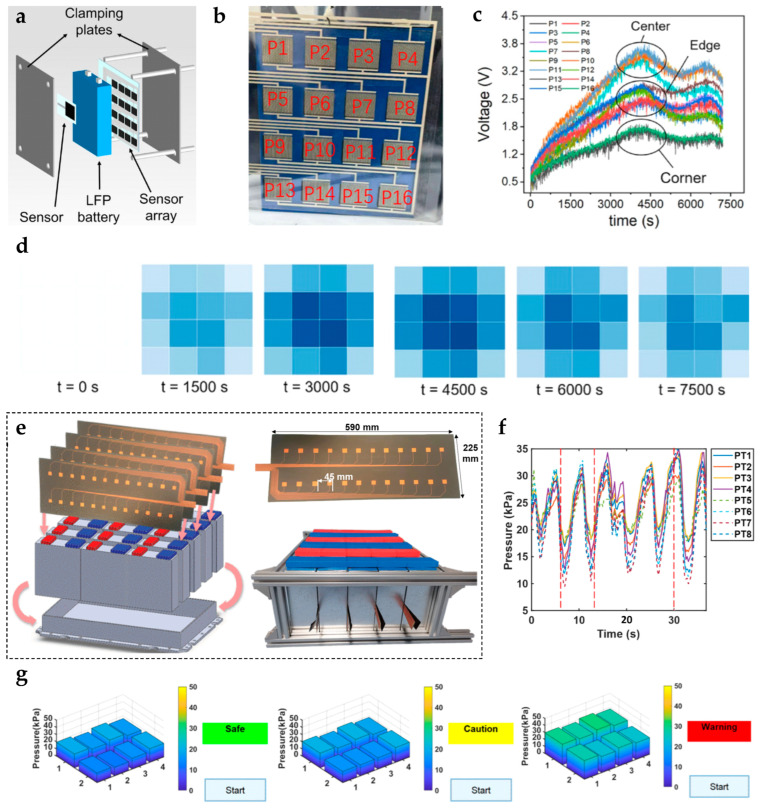
(**a**) Schematic of a lithium-ion battery testing setup integrated with a flexible pressure sensor array. (**b**) Image of the sensor array and the battery. (**c**) Analysis of the sensor array test results. Notably, transient force reversals are observed during charge and discharge processes. (**d**) Distribution of swelling force under different charge–discharge states of the battery [262]; © Wiley, 2025. (**e**) Schematic of a battery module integrated with a flexible pressure sensor array. The inset shows image of the sensor array and the battery module. (**f**) Analysis of the sensor array test results. (**g**) Three-dimensional visualization of different swelling states of the battery module [235]; © IEEE, 2025.

To address these limitations, array-based monitoring strategies have been introduced. Deploying flexible pressure sensor arrays on module surfaces or between cells achieves high spatial resolution and the dynamic evolution of expansion forces [262] (Figure 13a). The array conforms to the cell surface (Figure 13b), and its flexibility allows it to accommodate cell surface deformation. Then, simultaneous data acquisition from all units enables the mapping of expansion force distributions under different charge–discharge conditions (Figure 13c). Finally, 3D reconstruction algorithms generate spatial visualizations of module expansion (Figure 13d). For large prismatic modules, modular array designs can cover the entire module [235] (Figure 13e), enabling the production of combined expansion force curves (Figure 13f) and three-dimensional distributions under various states (Figure 13g). This reveals the spatial heterogeneity and evolution of module expansion.

In practice, mapping in 3D after embedding arrays between prismatic module cells revealed that, after 100 cycles, edge-region pressure decay was 15% higher than at the center [263]. This confirms the “edge-to-center” non-uniform expansion trend.

In summary, single-point and array-based monitoring methods have complementary advantages. The former is simple and cost-effective and is suitable for tracking overall trends and providing basic warnings. The latter is optimal for fine-grained analysis and fault localization in high-energy-density, complex modules due to its distributed spatial sensing. As modules evolve toward larger, more integrated designs, array-based monitoring is expected to provide higher spatial resolution and a faster dynamic response.

### 4.4. Smart Data Analysis for Lithium Batteries

In the preceding sections, we systematically reviewed the application of flexible pressure sensors for monitoring the swelling force of lithium-ion battery modules. While these approaches provide abundant swelling-force information, the growing complexity of battery systems makes it difficult for conventional data processing methods to accurately and efficiently assess battery state of health (SOH) and safety risks in real time. The introduction of artificial intelligence (AI) provides an effective means of intelligently analyzing and predicting swelling-force signals, thereby enhancing the value of flexible sensors in battery management systems (BMSs) [264,265].

**Figure 14:**
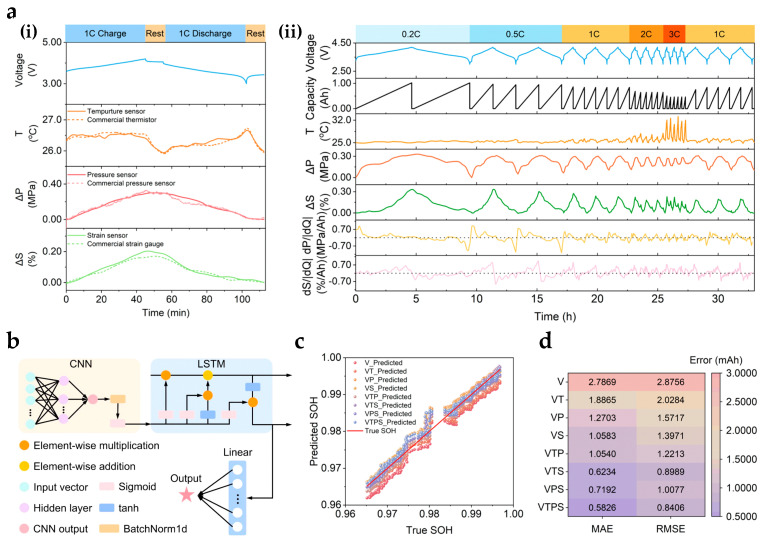
(**a**) Data for model training and analysis. (**i**) Characteristic data collected during charging and discharging of pouch batteries (1.1 Ah) at 25 °C using the integrated intelligent sensing array system (IISAS) and commercial sensors, including voltage, T, ∆P, and ∆S data; (**ii**) Characteristic data collected by IISAS at various charge/discharge rates on lithium batteries. (**b**) The framework of the deep-learning approach. (**c**) Validation results comparing the estimated and actual battery state of health (SOH) for the intelligent cells. (**d**) Averaged lowest mean absolute error (MAE) and root-mean-square error (RMSE) of the validation cells using different combinations of relevant features [16]; © Springer Nature, 2025.

As illustrated in Figure 14, multi-source data collected by flexible sensors, including voltage, temperature (T), swelling force (ΔP), and strain (ΔS), can be processed through feature engineering to derive indicators such as dP/d|Q| and dS/d|Q|, reflecting the coupled mechanical and electrochemical behavior of batteries at different states of charge (Figure 14a). Based on these original and derived features, deep learning frameworks can be developed (Figure 14b) to enable multidimensional data fusion and dynamic SOH prediction with real-time tracking capabilities (Figure 14c). Such models’ performance can be quantitatively evaluated using metrics such as mean absolute error (MAE) and root mean square error (RMSE). The influence of different feature combinations on prediction accuracy can also be systematically compared (Figure 14d) [16].

Furthermore, integrating AI with multi-sensor data has been proven to greatly enhance the accuracy of state estimation. Yang et al. [266] proposed a SOC prediction method based on extreme learning machines (ELM) combined with radial basis function neural networks (RBFNN). Their approach effectively captured the dynamic behavior of batteries under various charging and discharging conditions by incorporating strain signals acquired from fiber Bragg grating (FBG) sensors. This resulted in substantially reduced SOC estimation errors and provided valuable reference features for swelling-force analysis.

Meanwhile, Sun et al. [262] focused on real-time swelling-force monitoring and developed a shear-thinning ink-based flexible sensor. They also established a residual CNN-LSTM data-fusion framework that integrates voltage and swelling-force measurements. This approach achieved an unprecedented SOC prediction accuracy of 98.13% at a 1% resolution. The study demonstrated a practical solution for monitoring the mechanical state of batteries and confirmed the potential of swelling force as a key parameter in advanced battery management systems. So, this offers new strategies for predicting safety and estimating the state of energy storage applications.

In summary, AI-driven intelligent data analysis can detect subtle swelling behaviors, facilitate real-time fusion and pattern recognition of multi-source signals, and provide a robust foundation for predicting battery lifetime, enabling early warnings of safety issues, and optimizing charging and discharging strategies. With continued advances in sensor performance and computational capability, this approach is expected to become critical for the deep integration of flexible sensing technologies with intelligent battery management systems.

## 5. Current Challenges and Trends

### 5.1. Current Challenges

The application of flexible pressure-sensing technologies for monitoring battery swelling is rapidly transitioning from laboratory research to practical engineering. However, the complex operating conditions of batteries and the strict requirements for long-term, accurate monitoring pose significant challenges to the materials, structural design, sensing mechanism, sensing performance, and systems of these sensors.

At the material level, the main difficulty is achieving a balance of flexibility, stability, and electrochemical compatibility. Commonly used sensitive materials, such as carbon nanotube/elastomer composites and hydrogels, tend to swell, dehydrate, or degrade when exposed to electrolytes or high temperatures. These changes disrupt conductive networks and lead to progressive sensitivity deterioration. Electrode materials face similar constraints: metal thin films are susceptible to oxidation and delamination, and flexible conductive fabrics often exhibit unstable interfacial resistance. Encapsulation materials must isolate the sensor from harsh environments; however, their thermal expansion mismatch with battery casings can introduce parasitic stresses during thermal cycling. These stresses impair adhesion and may disturb the battery’s thermal management pathways.

At the structural level, challenges stem from geometric adaptation and integration compatibility. Different battery formats impose distinct mechanical requirements. For example, cylindrical cells require sensors with exceptional bending robustness to conform to curved surfaces. In contrast, prismatic-module configurations demand ultrathin sensors to fit the narrow inter-cell gaps without disrupting module assembly. Embedded sensor architectures are highly sensitive to placement errors or insufficient encapsulation; either could induce internal short circuits. On the other hand, surface-mounted sensors rely on fixtures or adhesive layers that may undergo stress relaxation, creep, or interfacial aging during long-term cycling. These effects undermine measurement reliability and make it difficult to meet the high lifetime requirements of commercial batteries.

At the mechanistic level, battery swelling arises from several concurrent processes, such as electrode lithiation, SEI growth, gas generation, and structural degradation. The contribution of each mechanism varies depending on the state of charge, temperature, cycling history, and aging. This makes it difficult to link measured swelling signals to specific internal processes quantitatively. Additionally, swelling responses usually consist of reversible and irreversible components. For example, elastic deformation that occurs during normal cycling overlaps with the irreversible expansion caused by SEI thickening, lithium plating, or gas accumulation. This complicates signal interpretation and limits the ability to distinguish healthy operation from early-stage degradation using pressure data alone. Additionally, the mismatch in timescales between electrochemical reactions and mechanical responses introduces further complexity. Gradual degradation processes exist alongside abrupt swelling events, and capturing these various temporal features under realistic operating conditions is highly challenging.

The primary limitation in performance is the inability to reliably monitor multiple scenarios throughout the entire battery lifespan. Dynamic and static sensing capabilities are often imbalanced. For example, piezoelectric and triboelectric sensors excel at capturing millisecond-scale transient swelling events, but they fail to support long-duration, steady-state measurements. Conversely, capacitive and resistive sensors are suitable for static monitoring, yet they respond inadequately to sudden bulging events. Environmental robustness is another major issue. Temperature fluctuations can strongly affect the electrical properties of sensitive materials, and humidity variations may introduce drift or offset. Furthermore, the cyclic mechanical loading inherent to battery operation causes progressive fatigue in the sensitive layer, reducing accuracy and hindering the ability to sustain full-lifecycle monitoring.

Beyond the sensing layer, system-level challenges remain equally significant. Large-scale flexible sensor arrays generate massive data streams, which creates substantial demands on transmission bandwidth, real-time processing, and power consumption. Meanwhile, the electrochemical behaviors of batteries are highly coupled and nonlinear. Swelling forces are influenced by SOC, solid-electrolyte interphase evolution, gas generation, increased internal resistance, and latent failure modes, such as thermal runaway. Because these relationships are complex and not linearly correlated, converting swelling signals into accurate battery health indicators (SOH estimation and early warning of internal faults) requires advanced modeling approaches, multi-physics data fusion, and intelligent diagnostic algorithms.

### 5.2. Future Development Trends

**Figure 15:**
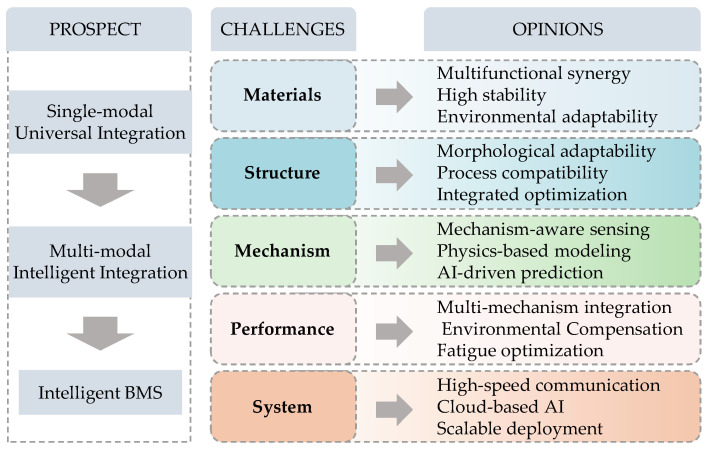
Future development trends in flexible sensing technology.

To overcome current challenges in materials, structural design, sensing mechanism, sensing performance, and system-level integration, future flexible pressure sensing technology development for battery swelling monitoring is expected to be more systematic, integrated, and intelligent (Figure 15).

Regarding material systems, future research will focus on multifunctional synergy and enhancing long-term stability. Introducing fluoride-based protective coatings and developing inorganic–organic hybrid hydrogel networks can substantially improve the corrosion tolerance and thermal stability of sensing materials in electrolyte-rich environments. Meanwhile, encapsulation materials will evolve to better accommodate the thermal expansion behavior of battery cells. Polyimide-based composite films combined with hydrophobic interfacial layers offer robust environmental isolation and low mechanical stress, ensuring stable sensor-cell adhesion during long-term cycling.

At the structural design level, the focus will be on morphological adaptability and process compatibility. For cylindrical cells, deformable structures such as helical or mesh-like flexible arrays, can provide high conformability and withstand repeated strain. For prismatic battery modules with narrow inter-cell gaps, ultrathin and high-toughness integrated designs are essential, in which serpentine interconnects and wrinkled architectures significantly enhance tensile and bending flexibility. From an integration perspective, embedded sensors can be coordinated with battery manufacturing processes. In contrast, surface-mounted sensors rely on reinforced adhesion layers and advanced Mechanical Clamps to ensure stable attachment throughout the battery’s service life.

At the mechanistic level, future development will focus on integrating understanding of the mechanisms into sensing, signal interpretation, and predictive frameworks. The goal is to correlate swelling signals with electrochemical and mechanical processes such as electrode lithiation, SEI evolution, gas generation, and irreversible structural changes. Physics-driven models and multiscale simulations can decouple reversible and irreversible swelling processes, enhancing interpretability and accuracy. Additionally, hybrid machine learning approaches that incorporate mechanistic priors are expected to improve predictive capabilities. These capabilities will enable accurate SOH assessments, early failure detection, and remaining useful life (RUL) predictions. Time-resolved monitoring and adaptive signal processing capture both gradual degradation and abrupt swelling events, thus bridging the temporal mismatch between electrochemical reactions and mechanical responses. Mechanism-informed sensor placement and structural optimization ensure that swelling measurements accurately reflect internal battery states while minimizing interference with electrochemical performance.

With regard to performance optimization, the primary objective is to achieve comprehensive monitoring across a range of operating scenarios. Future sensor systems will likely incorporate multiple sensing mechanisms, such as combined piezoelectric-capacitive designs, to simultaneously capture transient impacts and steady-state swelling. Embedded temperature compensation, low-noise signal conditioning, and adaptive environmental calibration algorithms enhance robustness against thermal and humidity variations. Additionally, simulation-driven structural optimization can improve the fatigue tolerance of the sensing layer, thus enabling reliable operation throughout the battery’s entire lifecycle.

At the system level, advancements will focus on efficient data transmission and AI-driven health assessments. High-speed communication technologies, such as 5G, will enable parallel, multi-channel data transfer from distributed sensing nodes to edge gateways, thereby improving bandwidth and real-time performance. Cloud computing platforms that integrate digital battery models and AI models will analyze massive, multi-parameter datasets, including swelling force, temperature, and voltage, to provide accurate state-of-health estimation, detect abnormal swelling patterns early, and predict remaining useful life (RUL) dynamically.

Overall, the coordinated progress across materials, structures, sensing mechanism, sensing performance, and system intelligence will accelerate the transition of flexible pressure sensing technologies from laboratory demonstrations toward engineered, scalable, and intelligent battery monitoring solutions.

## 6. Conclusions

The swelling behavior of lithium-ion batteries stems from a complex electrochemical-mechanical coupling process. Precise monitoring is essential to ensure operational safety and extend service lifetime. This work begins with a mechanistic analysis of battery expansion and provides a systematic review of existing monitoring techniques. These techniques range from conventional methods, such as dilatometry, buoyancy measurement, and strain-based approaches, to emerging technologies, including optical fiber sensing, radiographic imaging, optical interferometry, and electrochemical impedance spectroscopy.

Among these methods, flexible pressure sensing is exceptional due to its mechanical compliance, stretchability, and adaptability. These sensors can conformally integrate with various battery architectures, including pouch, cylindrical, and prismatic cells, enabling the accurate, real-time acquisition of expansion signals without disrupting normal electrochemical operation. Furthermore, sensor array designs allow flexible pressure sensors to achieve high spatial resolution mapping of expansion forces. This is particularly valuable for detecting localized abnormal swelling and supporting rigorous assessment of battery health.

Despite these advantages, the practical deployment of the technology still faces several challenges. These include the limited long-term stability of sensing materials, the insufficient suppression of environmental interference, and the difficulty of maintaining signal consistency across large-scale arrays. Future advances will require coordinated progress in materials science, structural engineering, sensing mechanism, and system-level integration. Promising efforts include developing next-generation sensing materials with enhanced electrochemical stability and thermal robustness, designing sensor architectures that are both fatigue-resistant and manufacturing-compatible, and integrating advanced data-fusion algorithms. With the rapid convergence of sensing technologies and artificial intelligence, intelligent flexible sensing systems that can simultaneously monitor multiple parameters and autonomously predict the health state are expected to become a key part of next-generation battery management systems (BMSs). These systems will lay a solid technological foundation for the safe and efficient management of lithium-ion batteries throughout their lifecycle.

## Figures and Tables

**Table 1:** Comparison of battery swelling mechanisms, category, characteristics, and effects.

	Mechanism	Category	Characteristics	Effects
Reversible swelling	Anode lithiation/delithiation	Electrochemical swelling	Strongly correlated with state of charge	Reflects electrochemical reaction states
	Thermal expansion	Thermo-mechanical swelling	Recovers with temperature variation	Induces structural degradation
Irreversible swelling	SEI layer thickening	Interfacial reaction-induced swelling	Accumulates progressively during cycling	Consumes active lithium; Increases internal resistance
	Lithium plating	Structural damage-induced swelling	Associated with safety risks	Causes irreversible volume expansion
	Electrolyte decomposition	Chemical reaction-induced swelling	Gas continuously buildup	Leads to internal pressure buildup and failure

**Table 2:** Comparison of different battery swelling monitoring methods.

	Methods	Advantages	Disadvantages	Refs.
Contact methods	Dilatometer	Low cost In situ measurement Nondestructive method	1D measurement Measurement on macro scale	[109]
	Buoyancy	Assess gas formation 3D measurement High resolution	Volume expansion monitoring Strict fluid selection	[117]
	Strain Gauge	Low cost Easy integration	Temperature drift Structural damage	[124]
	Optical Fiber Sensor	Easy integration Fast measurement acquisition	Requiring adhesive fixation Temperature drift	[131]
Non-contact methods	X-ray	Nondestructive method Microscopic structural assessment	Radiation safety concerns High cost Slow data acquisition	[137]
	Neutron	Nondestructive method Microscopic structural assessment Visualizes lithium transport	Radiation safety concerns High cost Slow data acquisition	[145]
	Laser Triangulation	Easy implement Flexible measurement solutions Low cost	Integration difficulties 3D measurement requires scanning Environmental interference	[150]
	White Light Interferometry	High resolution Fast data acquisition	High cost 3D measurement requires scanning	[157]
	3D Imaging	Simultaneous 3D measurement Flexible measurement solutions	High cost Resolution limited	[69]
	EIS-Based Evaluation	Simultaneous monitoring In situ dynamic monitoring	Poor specificity Difficulties in quantitative analysis Complexity of data post-processing	[163]

**Table 3:** Performance comparison of flexible pressure sensors with different mechanisms.

Performance	Capacitive	Iontronic	Resistive	Piezoelectric	Triboelectric
Sensitivity	1–100 kPa^−1^	100–10^4^ kPa^−1^	0.1–10 kPa^−1^	1–50 pC/N	10–100 V/kPa
Range	1 Pa–1 MPa	1 Pa–1 MPa	10 Pa–10 MPa	10 Pa–100 kPa	10 Pa–1 MPa
Hysteresis	<5%	<2%	5–20%	<1%	2–8%
Stability	10^4^–10^5^ cycles	10^3^–10^4^ cycles	10^5^–10^6^ cycles	10^6^–10^7^ cycles	10^4^–10^5^ cycles
Response Time	1–100 ms	0.1–100 ms	10–100 ms	<1 ms	1–100 ms
Environmental Immunity	Susceptible to humidity and temperature	Susceptible to ion environment state	Susceptible to temperature	Susceptible to temperature	Susceptible to humidity and temperature
Battery Applications	Strong adaptability High dynamic response	Weak swelling monitoring In situ monitoring	Wide-range monitoring Low cost	High-dynamic monitoring High-frequency capture	High self-powering ability

**Table 4:** Comparison of advantages and disadvantages between surface-mounted and embedded types.

	Advantages	Disadvantages	Application
Surface-Mounted	Simple installation Low cost	Significant hysteresis Limited accuracy	Consumer electronics Offline testing
Embedded	Highest accuracy No hysteresis	Destroys cell structure High safety risk	Scientific research Custom batteries

**Table 5:** Comparison of the advantages and disadvantages of different integration strategies.

	Advantages	Disadvantages	Application
Adhesive	Fast deployment low cost high adaptability	Susceptible to interfacial aging and temperature effects Limited to surface information	Surface monitoring of single cells/modules rapid prototyping
Fixture	Stable mechanical coupling High long-term measurement accuracy	Larger structural space Disrupting module assembly	Module assembly long-term cycling tests
Implant	Captures internal strain High spatial–temporal resolution	Interference Structure Design Complex embedding process	Highly integrated modules In situ Monitoring System

## Data Availability

No new data were created.
